# A new inhibitor of glucose-6-phosphate dehydrogenase blocks pentose phosphate pathway and suppresses malignant proliferation and metastasis in vivo

**DOI:** 10.1038/s41419-018-0635-5

**Published:** 2018-05-14

**Authors:** Luigi Mele, Francesca Paino, Federica Papaccio, Tarik Regad, David Boocock, Paola Stiuso, Angela Lombardi, Davide Liccardo, Gabriella Aquino, Antonio Barbieri, Claudio Arra, Clare Coveney, Marcella La Noce, Gianpaolo Papaccio, Michele Caraglia, Virginia Tirino, Vincenzo Desiderio

**Affiliations:** 1 Department of Experimental Medicine, University of Campania “Luigi Vanvitelli”, 80138 Naples, Italy; 2 Oncologia Medica ed Ematologia, Dipartimento Medico-Chirurgico di Internistica Clinica e Sperimentale “F. Magrassi e A. Lanzara”, University of Campania “Luigi Vanvitelli”, 80138 Naples, Italy; 3 The John van Geest Cancer Research Centre, School of Science and Technology, Nottingham Trent University, Clifton Lane, NG1 4FQ Nottingham, UK; 4 Department of Biochemistry, Biophysics and General Pathology, University of Campania “Luigi Vanvitelli”, 80138 Naples, Italy; 5 Department of Research, Pathology Unit, Istituto Nazionale Tumori- IRCCS- Fondazione Pascale, 80131 Naples, Italy; 6 SSD Sperimentazione Animale, Istituto Nazionale Tumori- IRCCS- Fondazione Pascale, 80131 Naples, Italy

## Abstract

Pentose phosphate pathway (PPP) is a major glucose metabolism pathway, which has a fundamental role in cancer growth and metastasis. Even though PPP blockade has been pointed out as a very promising strategy against cancer, effective anti-PPP agents are not still available in the clinical setting. Here we demonstrate that the natural molecule polydatin inhibits glucose-6-phosphate dehydrogenase (G6PD), the key enzyme of PPP. Polydatin blocks G6PD causing accumulation of reactive oxygen species and strong increase of endoplasmic reticulum stress. These effects are followed by cell cycle block in S phase, an about 50% of apoptosis, and 60% inhibition of invasion in vitro. Accordingly, in an orthotopic metastatic model of tongue cancer, 100 mg/kg polydatin induced an about 30% tumor size reduction with an about 80% inhibition of lymph node metastases and 50% reduction of lymph node size (*p* < 0.005). Polydatin is not toxic in animals up to a dose of 200 mg/kg and a phase II clinical trial shows that it is also well tolerated in humans (40 mg twice a day for 90 days). Thus, polydatin may be used as a reliable tool to limit human cancer growth and metastatic spread.

## Introduction

The pentose phosphate pathway (PPP) has recently been shown to have a crucial role in cancer cell growth by providing both nucleotide precursors, needed for proliferation, and NADPH used for both intracellular ROS detoxification and catabolic metabolism^1–5^. The inhibition of PPP key enzymes, including glucose-6-phosphate dehydrogenase (G6PD), strongly affects cancer cell proliferation in vitro, as well as in vivo^6, 7^. G6PD is upregulated in many human cancers and correlates with poor prognosis^8–12^, whereas cancer patients harboring G6PD mutation show longer survival and reduced metastases^13–15^. Moreover, G6PD activity can be regulated by oncogenes such as PI3k/AKT, Ras, Src, mTORC1, or by oncosuppressors such as p53 and TAp73^4^. Interestingly, the inhibition of G6PD may restore sensitivity of cancer cells to chemotherapy^16^. Therefore, PPP inhibition has been proposed as an attractive therapeutic strategy against cancer^17^. However, the inhibition of G6PD in clinical settings is hampered by the lack of specific inhibitors. To our knowledge, the only G6PD inhibitor ever used in vivo is the dehydropiandrosterone (DHEA), an endogenous steroid hormone, which is produced by adrenal glands acting as a metabolic precursor of androgen and estrogen. DHEA is rapidly converted into steroid hormones in vivo and its efficacy as an inhibitor of G6PD is under dispute^18^.

Polydatin (3,4′,5-trihydroxystilbene-3-β-d-glucoside; trans-resveratrol 3-β-mono-D-glucoside; piceid) is a natural molecule found in *Polygonum cuspidatum* and other plants. Polydatin is a glucoside of resveratrol and, together with other polyphenols, has been shown to have several biological effects, including the induction of apoptosis in carcinoma cells^19–22^. Here we have studied the effects of polydatin on G6PD activity, ROS levels, ER stress, and programmed cell death, and its role in inhibiting cancer cell proliferation and invasion both in vitro and in vivo.

## Results

### Polydatin inhibits cancer cell proliferation and cell cycle progression

**Fig. 1:**
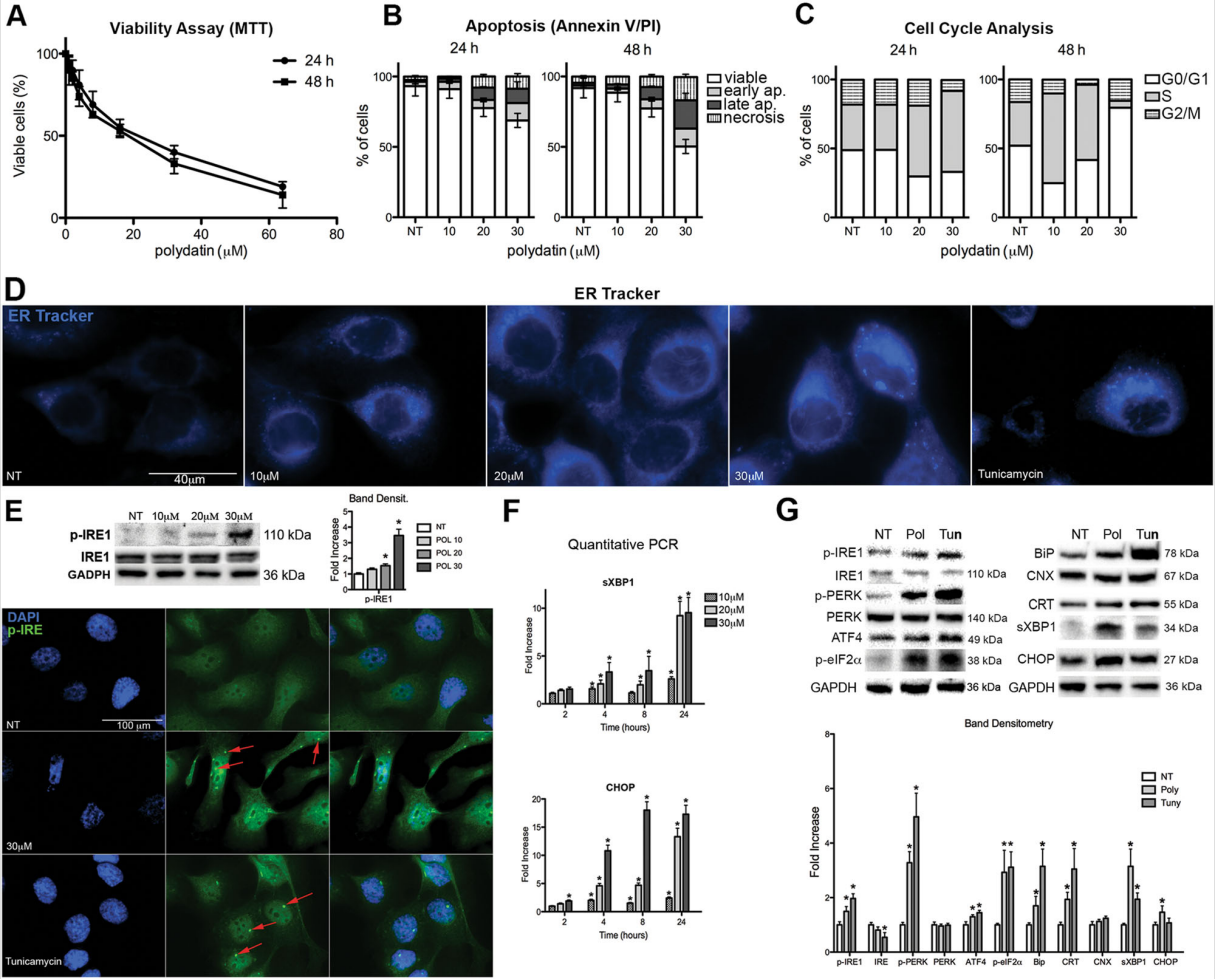
Effects of polydatin treatment on UMSCC103. **a** Viability assay measured by MTT (concentration range 0–70 µM) at 24 and 48 h posttreatment. **b** Analysis of apoptosis by Annexin V/PI assay at 24 and 48 h posttreatment (for original dot plots see Fig. S1A). **c** Cell cycle analysis (for original histograms and analyses see Fig. S1B). **d** IF with ER-Tracker 24 h posttreatment; tunicamycin is used as positive control. **e** Immunoblot for phospho-IRE1, 24 h posttreatment, polydatin treatments 10–30 μM. IF for phospho-IRE1, 24 h posttreatment; tunicamycin is used as positive control; red arrows represent phospho-IRE cluster. **f** Quantitative real time PCR for CHOP and spliced XBP1. **g** Immunoblot for UPR pathway and ER chaperons, 24 h posttreatment, Polydatin 20 μM. *p < 0.05. *N* = 3, error bar = 95% confidence

We assessed the viability of head and neck squamous cell carcinoma (HNSCC) cell lines after polydatin treatments at different concentrations (from 2 to 100 µM at 24 or 48 h), by MTT assay. We found that polydatin-reduced cell viability in a dose- and time-dependent manner at an EC50 of 22 µM for 24 h and 17 µM for 48 h, respectively (Fig. 1a). Based on these data we have selected, for all the subsequent experiments, concentrations of 10, 20, and 30 µM that represent the EC25, EC50, and EC75. To confirm the effects on cell viability, we performed an apoptosis assay based on Annexin V/PI staining (Fig. 1b and S1A). We observed a dose- and time-dependent reduction of cell viability and an increased apoptosis and necrosis of treated cells. Polydatin affect, as well the cell cycle inducing a block in the S phase that reflected the ability of polydatin to inhibit cell proliferation (Fig. 1c and Fig. S1B). These results demonstrate that polydatin reduces viability, increases apoptosis, and prevents cell cycle progression of primary HNSCC cells. Similar results were obtained on breast cancer MCF7 cell line (Fig. S2D-F).

### Polydatin induces ER stress-driven cell death

**Fig. 2:**
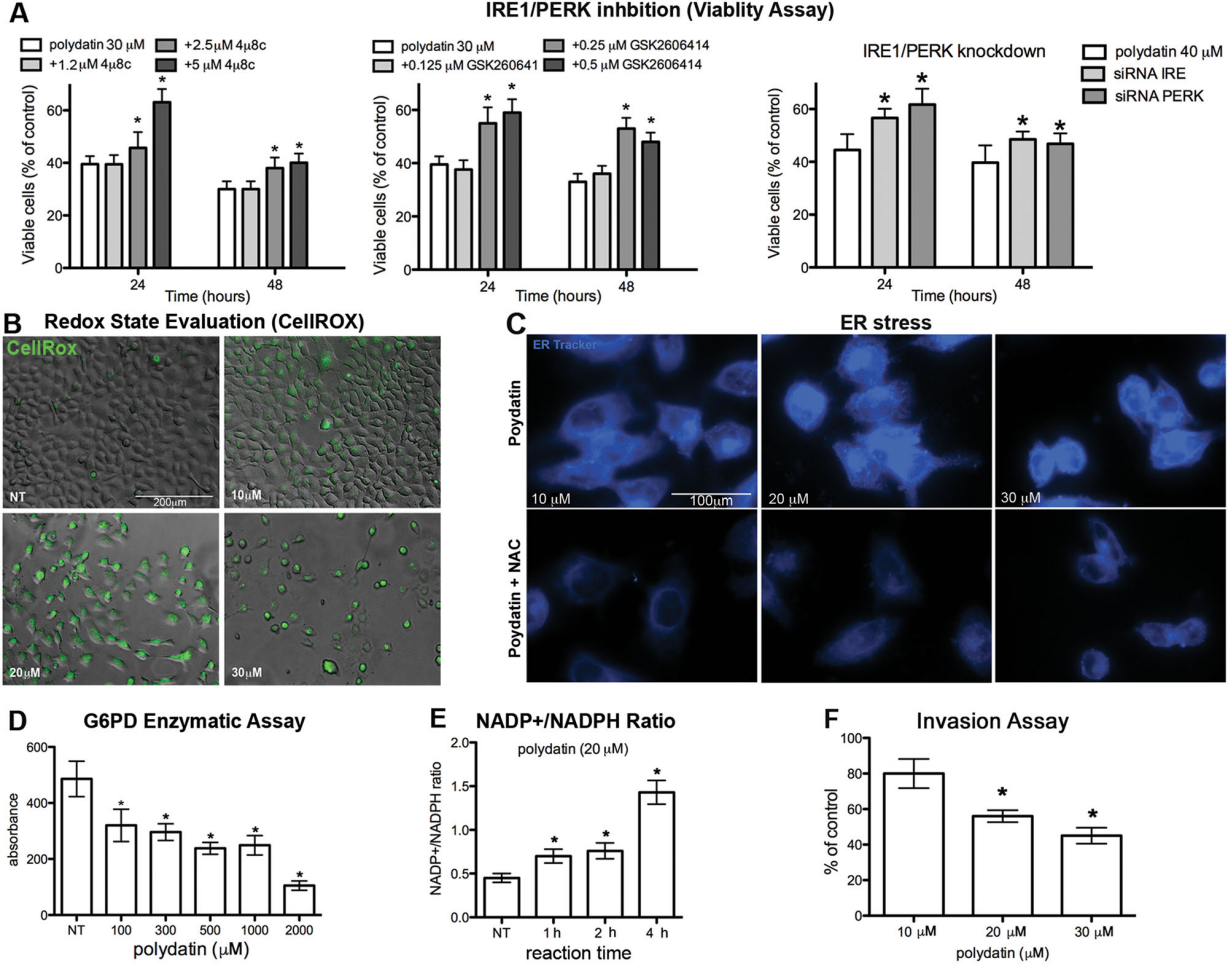
Polydatin Inhibits G6PD causing redox imbalance, which leads to ER stress and cell death. a Viability assay (MTT) of UMSCC103 treated with polydatin (20 µM) and 4μ8c (IRE inhibitor), GSK2606414 (PERK inhibitor) or with siRNA for either IRE1 or PERK, 24 or 48 h posttreatment, respectively. **b** IF with CellROX (for oxidative stress determination), 24 h posttreatment (In fig S5: menadione is used as positive control; *N*-acetylcysteine (NAC) is used as ROS scavenger; quantitative assay). **c** IF with ER-Tracker on UMSCC103 treated with polydatin in combination with NAC, 24 h posttreatment. **d** G6PD enzymatic assay on UMSCC103 cell lysates (the same assay performed with purified enzyme is found in Fig. S2C). **e** NADP + /NADPH ratio on polydatin-treated cells. **f** Invasion assay of UMSCC103 after polydatin treatment. *p < 0.05, *N* = 3, error bar = 95% confidence

To understand the molecular mechanism leading to cell death, we hypothesized that polydatin could cause endoplasmic reticulum (ER) stress, which in turn can lead to apoptosis. We performed an immunofluorescence staining (IF) using the vital dye ER-Tracker, which accumulate into ER. As a positive control, we used tunicamycin, a known inducer of ER stress. Following treatment with either tunicamycin or different concentrations of polydatin, we observed an increased fluorescence, which corresponds to an enlarged ER (Fig. 1d). Inositol-requiring enzyme 1 (IRE1) is an enzyme with intrinsic kinase and endoribonuclease activity that is activated during ER stress. After oligomerization and auto-phosphorylation, IRE1 acts on X-box binding protein 1 (XBP1) mRNA and causes an unconventional alternative splicing that activates XBP1 transcription factor to upregulate ER chaperones. Clusters of oligomerization of phospho-IRE1 were observed in both polydatin and tunicamycin-treated cells by both IF (Fig. 1e) and immunoblotting (Fig. 1f). Moreover, a significant increase of XBP1 mRNA and its spliced form (sXBP1 mRNA) was observed. This effect was dose- and time-dependent and began 4 h following treatment (Fig. 1f). Following ER stress, both IRE1 and PERK (protein kinase RNA-like ER kinase) induce the transcription of CCAAT-enhancer-binding protein homologous protein (CHOP), which is involved in the activation of apoptosis. As expected, the phosphorylated form of PERK and two of its downstream effectors p-eIF2α and ATF4 increased after polydatin treatment (Fig. 1g). Finally, CHOP transcript and protein also increased in response to polydatin treatment in a dose- and time-dependent manner as early as 2 h after treatment (Fig. 1f, g). ER chaperons such as BiP and CRT increased after treatment too, while CNX did not show significant changes upon treatment. To understand whether ER stress was driving cell death, we showed that the block of IRE1 or PERK activation, by specific inhibitors or by knockdown, partially reverted polydatin-induced cytotoxicity (Fig. 2a).

### Polydatin inhibits G6PD and induces oxidative and ER stresses

To additionally investigate the molecular mechanism of action of polydatin, we performed a mass spectrometry protein expression analysis of cell lysates from untreated and polydatin-treated cells (Fig. S2A and S2B) that was compared with one from tunicamycin-treated cells. As expected, the expression of proteins involved in ER stress was affected by tunicamycin treatment (Table S1). Polydatin-treated cells also showed a pattern of expression that was consistent with stress response and which overlapped that one of tunicamycin-treated cells. Among the proteins modulated by polydatin, we found a group of proteins belonging to the family of oxidoreductases and that were not altered in tunicamycin-treated cells (Fig. S2B and Table S1). Specifically, we found that seven oxidoreductases, including isocitrate dehydrogenase 1 (IDH1), G6PD and 6-phosphogluconate-dehydrogenase (6PGD), were significantly upregulated in polydatin treated samples (24 h treatment). G6PD and 6PGD are both part of PPP and together with IDH1 are responsible for the production of almost all the cytosolic NADPH, which is necessary for redox balance. Indeed, we found a significant reactive oxygen species (ROS) accumulation following polydatin treatment (Fig. 2b, S5). ROS plays a critical role in many cellular processes and can be produced in the cytosol and in several organelles, including ER and mitochondria. To determine the role of ROS accumulation in ER stress, we incubate the cells with the antioxidant *N*-acetylcysteine (NAC), prior to polydatin treatment. Pre-treatments with NAC completely abrogated ER stress induced by polydatin, suggesting a causative link between oxidative and ER stress (Fig. 2c).

As G6PD is the PPP limiting enzyme, its inhibition would affect NADPH production, and results in redox imbalance. Thus, we hypothesized that the biological effects observed could be a consequence of G6PD block. Therefore, we performed a G6PD enzyme activity assay on carcinoma protein lysates, as well as on purified G6PD. In both cases, polydatin-inhibited G6PD activity in a concentration-dependent manner (Fig. 2d, S2C). This was paralleled by an increase of the NADP + /NADPH ratio in a time-dependent manner (Fig. 2e). These results suggest that polydatin inhibits G6PD causing an imbalance in NADP + /NADPH ratio that leads to an increase of oxidative stress. The capacity of tumor cells to control oxidative stress through NADPH production is directly correlated with their ability to migrate. Indeed, in vitro invasion assays confirmed that polydatin-inhibited cancer cell invasion in a dose-dependent manner (Fig. 2f).

### G6PD overexpression counteracts polydatin antitumor effects

**Fig. 3:**
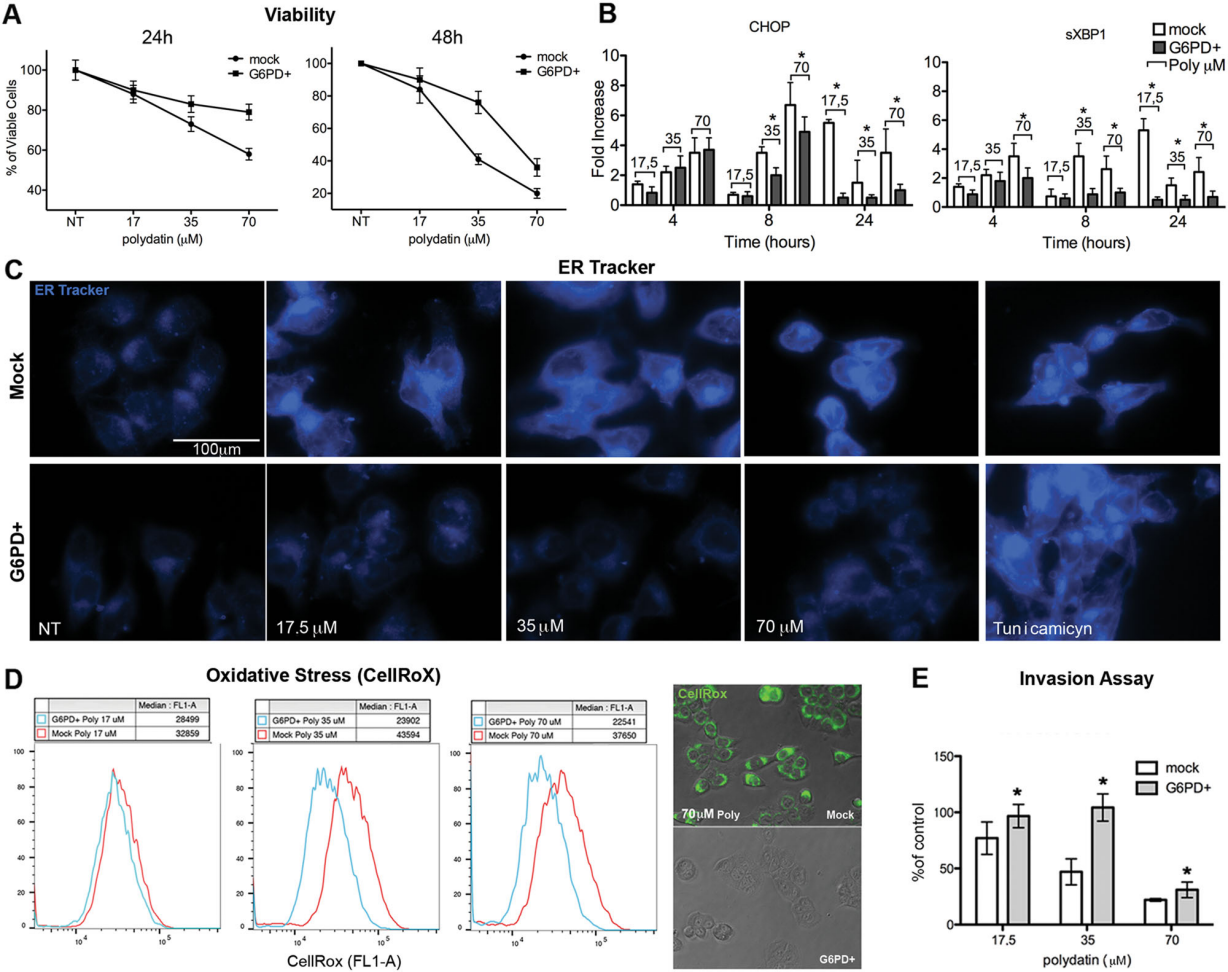
Generation of G6PD overexpressing cell lines. Differences with mock transfection after polydatin treatment. **a** Viability assay (MTT) in G6PD+ and mock-transfected cells following polydatin treatment. **b** Quantitative real time PCR on G6PD+ and mock-transfected cells for CHOP and spliced XBP1 after polydatin treatment**. c** IF with ER-Tracker on G6PD+ and mock-transfected cells after polydatin treatment. **d** Flow cytometry of CellRox (oxidative stress determination) on G6PD+ and mock-transfected cells following polydatin treatment. **e** Invasion assay in G6PD+ and mock-transfected cells. *p < 0.05, *N* = 3, error bar = 95% confidence

To confirm that G6PD inhibition was functionally related to the biological effects induced by polydatin, we generated a G6PD overexpressing cell line (MCF7^G6PD+^) (Fig. S3A). MCF7^G6PD+^ were resistant to polydatin treatment in both viability assay and apoptosis analyses. Indeed, 70 µM polydatin at 24 h cause a 50% reduction in viability in mock cells vs 35% in MCF7^G6PD+^. Moreover, 35 µM polydatin at 48 h caused 65% mortality in mock vs 40% in MCF7^G6PD+^ (Fig. S3B). Polydatin treatment caused a strong and significant increase of the S phase of cell cycle at 17.5 µM (16 ± 5% to 63 ± 10%), while it increased both S and G1 phase at the expense of G2/M at 35 and 70 μM in mock-transfected cells at 48 h. Analysis of cell cycle at 48 h showed always an increase of S and G2/M phases. On the other hand, the MCF7^G6PD+^ cell cycle, was much less perturbed by polydatin that induced only a small increase of S phase at a concentration of 35 µM at 48 h (from 20 ± 5% to 48 ± 6%) (Fig. S3C). MCF7^G6PD+^ cells were resistant to ER stress induced by polydatin as showed by ER-Tracker staining (Fig. S3D). On the other hand, tunicamycin induced a strong ER stress in both mock and MCF7^G6PD+^ cells. This result suggests that MCF7^G6PD+^ are not resistant to ER stress induced by mechanisms independent from redox imbalance. To further confirm this, we show that CHOP and spliced-XBP1 transcripts were significantly higher in polydatin-treated mock cells than MCF7^G6PD+^ (Fig. 3b). The phosphorylation and clustering of IRE1 was strong and evident in mock cells but completely undetectable in MCF7^G6PD+^ (Fig. S3D). Analysis of redox status after polydatin treatment revealed an about two-fold increase of ROS accumulation in mock cells at concentration above 17.5 µM (Fig. S3D) but no effect in MCF7^G6PD+^. Again, invasion ability of MCF7^G6PD+^ was in the same way much less inhibited by polydatin treatment if compared to mock cells (Fig. S3E).

### Polydatin prevents lymph node metastases in an experimental orthotopic model of cancer

**Fig. 4:**
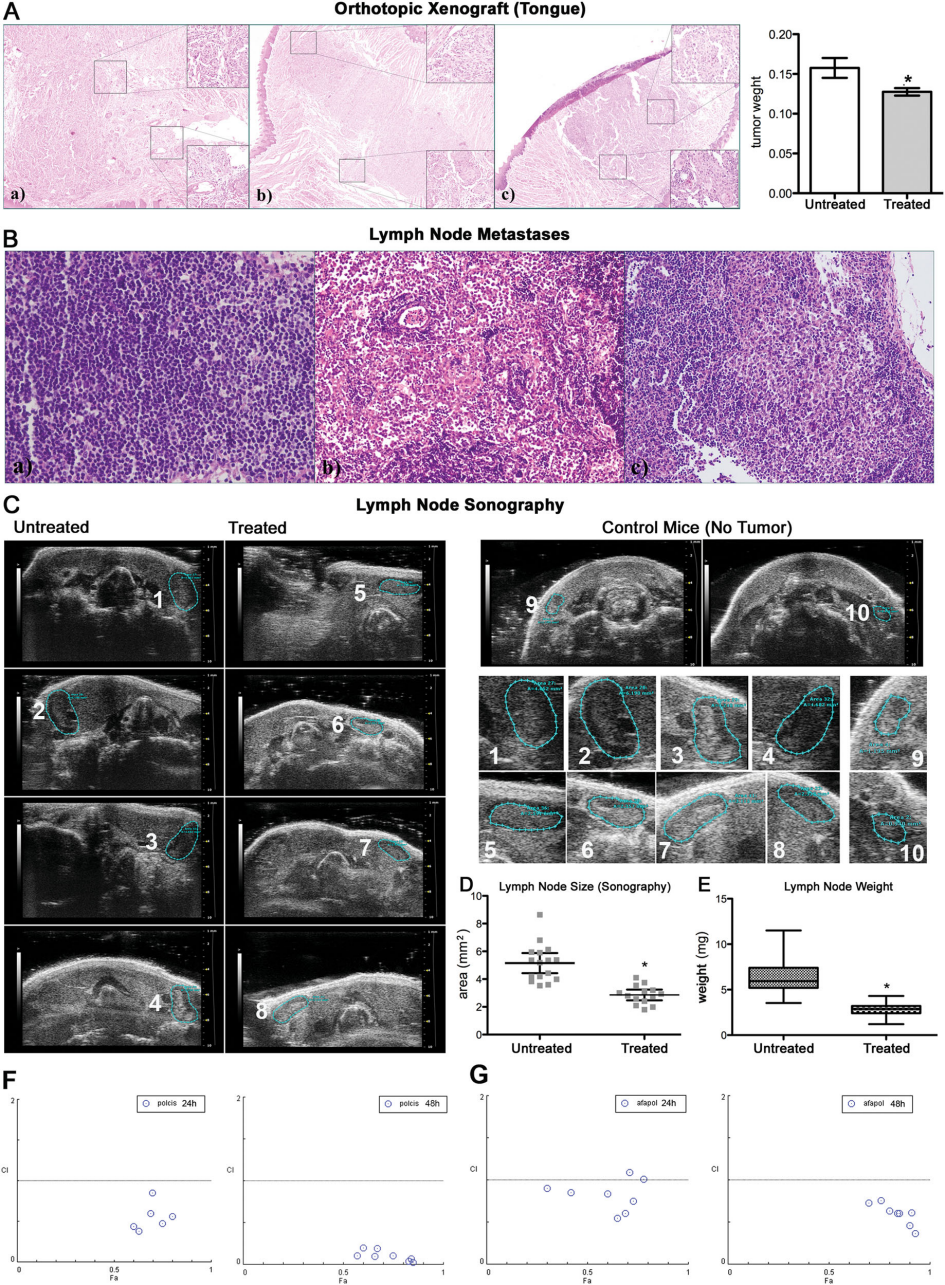
Analysis of tumor growth and lymph node invasion in an orthotopic xenograft model. **a** H&E staining of orthotopic xenograft in the tongue; bars, tongue weight of treated and untreated mice. **b** H&E staining of lymph node metastases. **c** Lymph node sonography and size determination on treated, untreated and control (no tumor) mice. Insets on the right, enlarged images. **d** Plot of lymph node size determined by sonography. **e** Lymph node weights of treated and untreated mice. **f**, **g** Analysis of combination index (CI) with compusin software for the combination polydatin/cisplatin and polydatin/afatinib. *p < 0.05, *N* = 3, error bar = 95% confidence

Polydatin-induced cancer cell death and blocked invasion in vitro. Therefore, we developed an orthotopic metastatic model of oral cancer by injecting UMSCC103 in mice tongues, assessing the timing of tumor growth and lymph node metastases occurrence. Primary tumors were examined at the end of the experiment when mice were killed, while lymph node metastases were followed using ultrasound imaging. Primary tumor growth was determined by weighting entire tongues and showed statistically significant differences between polydatin-treated and -untreated mice (Fig. 4a). The reduction of primary tumors’ growth was ≤30%. The squamous cell carcinoma nature of the implanted tumor was confirmed by an expert pathologist (Fig. 4a). Ultrasound imaging and size determination of cervical lymph nodes revealed a much greater effect of polydatin on metastases (Fig. 4c, d). In details, the average size of untreated mice lymph nodes was 5 ± 0.5 mm^2^ vs 3 ± 0.3 mm^2^ (*p* < 0.005) for the treated ones. These data were confirmed after mice killing and when lymph nodes were removed, weighted, and examined by a pathologist. We were able to identify and remove about 5/6 cervical lymph nodes per mouse. Indeed, the average weight of untreated mice was 6.2 ± 0.3 mg vs 2.9 ± 0.1 mg for the treated ones (*p* < 0.005) (Fig. 4e). All lymph nodes were then examined for metastases and we found that only two of the treated mice (out of ten) had lymph node metastases, while eight out of ten were observed in untreated ones.

### G6PD inhibition increase the effect of chemotherapy

PPP has been suggested to be involved in drug resistance in both solid tumors and leukemia. Two major work of Gregory et al.^23^ and Catanzaro et al.^16^ describe an involvement of G6PD in the resistance to cisplatin and tyrosine kinase inhibitor. For this reason, we performed analysis of synergism for the combination of cisplatin/polydatin and afatinib/polydatin. Both combinations were highly synergic at all the concentration used (Fig. 4f, g; S4).

## Discussion

In the present manuscript, we have studied the effect of polydatin on cancer cells elucidating its biochemical mechanism of action and biological effects. Based upon our results, we provide evidence showing that polydatin induces a potent cancer cell growth inhibition paralleled by a strong reduction of invasive properties of cancer cells in vitro and in vivo. The latter effect was reported for the first time and represents a major goal of cancer research, as cancer metastases control is an unmet need in cancer treatment. We demonstrate that polydatin directly inhibits G6PD, the limiting enzyme of PPP, causing redox imbalance, which results in ER stress, cell cycle arrest, and apoptosis. To elucidate the molecular mechanism of polydatin, we performed a quantitative proteomic analysis of lysates from polydatin-treated cells at multiple time points, and compared the protein expression profiles with tunicamycin-treated cells. This approach enabled the identification of a group of enzymes that significantly increased following the treatment with polydatin but not with tunicamycin. The enzymes belonged to the family of oxidoreductases and among these, three enzymes drew our attention: IDH1, G6PD, and 6PGD. These three enzymes are involved in glucose metabolism and account for the production of most of the NADPH. The latter has been proposed as rate limiting for cell proliferation^24–26^. Moreover, NADPH has been demonstrated to be fundamental for cancer growth and metastases^27, 28^. Indeed, we found that polydatin inhibited G6PD in either a cell lysate or using the purified enzyme. To demonstrate that the cytotoxic effects of polydatin were mediated by G6PD inhibition, we generated a cell line overexpressing G6PD. In these cells, treatment with polydatin resulted in a much lower effect if compared to control cells. This was observed for all the biological parameters, we analyzed. G6PD inhibition blocks proliferation and reduces DNA synthesis^29^, an effect similar to that one observed when cancer cells are treated with polydatin that inhibits cell cycle at the S phase^30^. In addition, the accumulation of cells in S phase of cell cycle at the expenses of G2/M is consistent with nucleotides shortage^31^. It has to be highlighted that, although polydatin has been used for more than 30 years, the molecular mechanism responsible for the effects reported have never been elucidated. We showed, for the first time, that the biological effects produced by polydatin treatment depend on G6PD inhibition and PPP block. This is, in our opinion, a pivotal finding, as specific G6PD or PPP inhibitors are not currently available in clinical setting. Indeed, although the PPP has been identified as a target for cancer therapy^4, 7, 9, 17, 24^ and recently pointed out to be responsible for drug resistance in humans by a relevant study of Gregory et al.^23^, the lack of specific inhibitors hampers research and clinical application that aim to target this pathway. The widely used G6PD inhibitor DHEA is rapidly converted in vivo into other hormones, which makes this drug not active^18^. The inhibitory effect recorded in some clinical trials seems to be more luckily produced by the interaction between DHEA and estrogen receptors. Moreover, the results obtained with DHEA in vitro need to be interpreted very carefully due to potential biasing by the several biological effects of this hormone. Interestingly, the capacity of tumor cells to control oxidative stress through NADPH production is directly correlated with their ability to form metastases in vivo^32^. Moreover, Richardson et al.^33^, used breast epithelial MCF10A cells (the parental and benign MCF10A, premalignant MCF10AT, and malignant MCF10CA1a) and showed that PPP flux increases with malignancy and correlates with tumor aggressiveness^33^. Consistently, the PPP is associated with invasiveness and seems to play a crucial role during the metastatic process by protecting metastatic cells^5, 34^. Therefore, our findings demonstrate that polydatin inhibits cancer cell invasion through PPP-dependent NADPH decrease. On the bases of these findings, we developed an orthotopic and metastatic model of oral cancer showing that polydatin strongly reduced both tumor growth and lymph node metastases. Interestingly, polydatin has previously been administered in different animal models to a dose up to 200 mg/kg with no reported major cardiovascular, hepatic, bone marrow, and renal toxicities^35–37^. Pharmacokinetic studies showed that polydatin is absorbed and distributed to tissues if given intravenously or by oral administration^38^. Phase II clinical trials have been performed using polydatin at 20–40 mg twice a day for a period, as long as 3 months and none of those reported any major cardiovascular, hepatic, bone marrow, and renal toxic effects^39, 40^. We showed that the combination of polydatin with either cisplatin or afatinib is strongly synergic in inducing cytotoxicity on cancer cells. These results strongly suggest the use of polydatin in clinical trials in combination with other antitumor agents in integrated anticancer strategies.

**Fig. 5:**
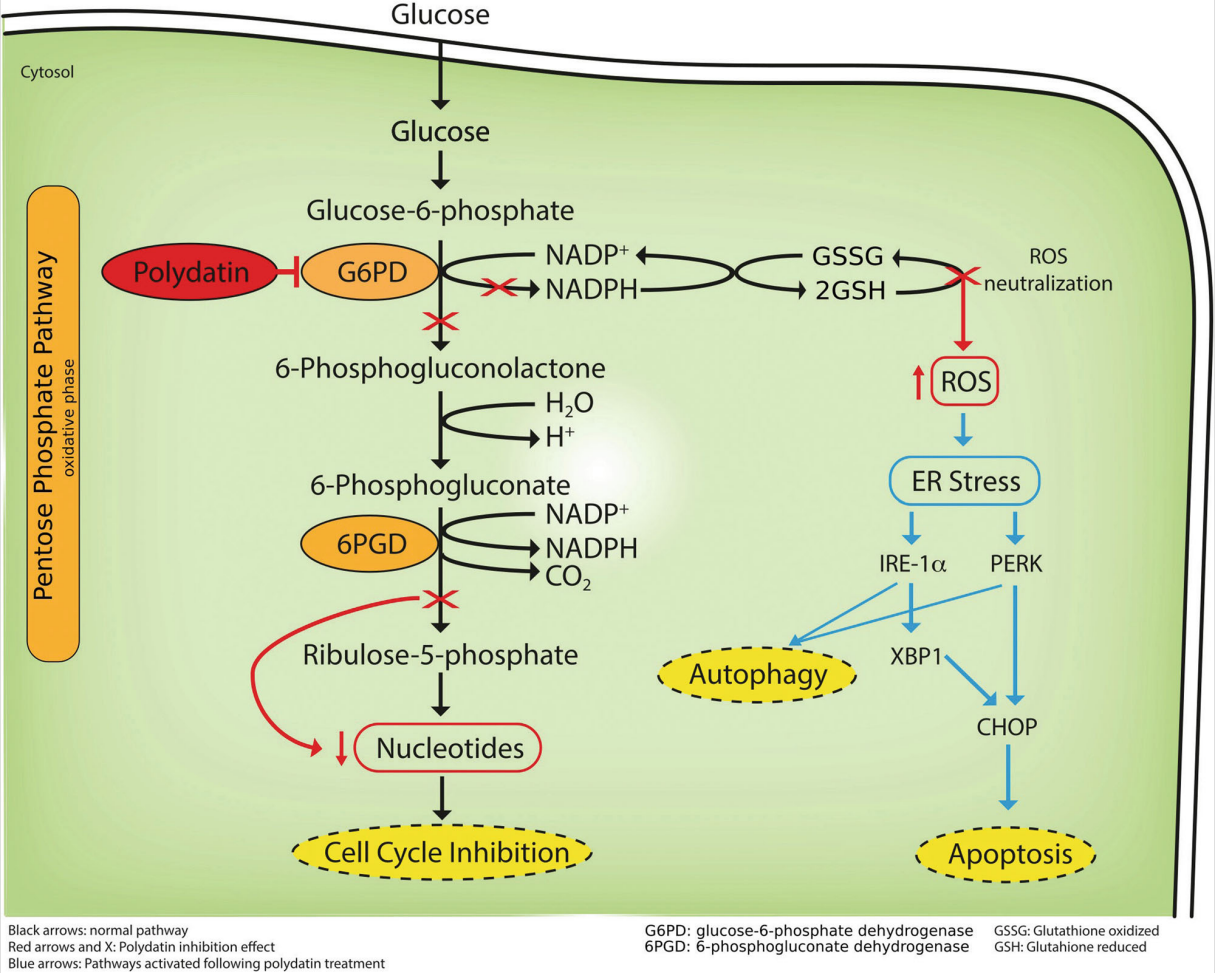
Schematic representation of Polydatin action on cancer cells

In conclusion, we show that polydatin reduces tumor growth and strongly inhibits lymph node metastases in oral cancer in vivo models with no toxicity. This effect has to be correlated with the direct inhibition of G6PD, the limiting enzyme of the PPP. This causes an impairment of NADPH production, causing ROS-mediated ER stress, apoptosis, and invasion inhibition (Fig. 5).

## Methods

### Chemicals, cell culture, and in vitro treatment

All chemicals were purchased from Sigma-Aldrich (St. Louis, USA) unless otherwise specified. Trans-polydatin, with a purity grade higher than 99%, was been supplied by Ghimas spa (Casalecchio, Bologna, Italy). Selective inhibitors of IRE1α (4μ8C) and PERK (GSK2606414) were obtained from Tocris Bioscience (Bristol, United Kingdom). Afatinib (BIBW2992) was obtained from Boehringer Ingelheim (Milano, Italy). UMSCC103 (OSCC cell line) used in this study were established at the University of Michigan under a protocol approved by the Institutional Review Board Office in accordance with the university’s regulations and described here^41^. MCF7 cells were purchased from ATCC. Cells were cultured in DMEM (Gibco, NY, USA) supplemented with 2 mM glutamine, 100 IU/mL penicillin, 100 μg/mL streptomycin (Invitrogen, Carlsbad, CA), and 10% heat-inactivated fetal bovine serum (FBS) (Gibco, NY, USA) at 37 °C in a humidified atmosphere under 5% CO_2_. All cell lines were kept mycoplasma free, checking was performed every 3 months.

### Invasion assay

The invasion assays were performed using a BD BioCoat Matrigel Invasion Chamber and BD control inserts (Becton-Dickinson, USA). The cells, exposed for 24 h to DMEM alone and with different concentrations of polydatin, were resuspended in serum-free DMEM and then added onto inserts with uncoated filters (control inserts) or onto inserts with Matrigel coated filters (invasion chambers) at the density of 5 × 10^4^ viable cells/insert. DMEM containing 10% FBS was used as the chemoattractant. Non-invading cells on the upper side of the membrane, after incubation for 18 h at 37 °C, 5% CO_2_, were removed with cotton swabs and the invading cells were fixed using methanol and stained with hematoxylin. Cells that invaded were counted at ×200 magnification. Each assay was performed in triplicate. Cells that invaded the Matrigel insert was compared to the number of cells that migrate in the control insert, calculating as percentage of invasion^42^.

### RNA isolation and qRT-PCR

Total RNA was isolated by RNeasy Mini Kit (Qiagen) according to manufacturer’s instructions, RNA was treated with DNase (Promega, Milan, Italy) to exclude DNA contamination and 1 μg total RNA reverse-transcribed using VILO SuperScript (Invitrogen, Monza, Italy). Gene expression assays were performed on a StepOne Thermocycler (Applied Biosystems, Monza, Italy) and the amplifications carried out using SYBR Green PCR Master Mix (Applied Biosystems, Monza, Italy). The reaction conditions were as follows: 95 °C for 15 min, followed by 40 cycles of three steps consisting of denaturation at 94 °C for 15 s, primer annealing at 60 °C for 30 s, and primer extension at 72 °C for 30 s. A melting curve analysis was performed from 70 °C to 95 °C in 0.3 °C intervals. Each sample was performed in triplicate. Glyceraldehyde 3-phosphate dehydrogenase (GAPDH) was used to normalize for differences in RNA input. qRT-PCR primer sequences are given in Supplementary Table 2.

### G6PD assay and NADP^+^/NADPH quantification

G6PDH activity was measured by Glucose 6 Phosphate Dehydrogenase Assay Colorimetric Kit (Abcam, Cambridge, UK). UMSCC103 cells untreated or treated with polydatin were homogenized in cold PBS, followed by centrifugation at 8000 × *g* for 10 min to remove insoluble materials. G6PDH activity was determined by analysis of G6PDH-dependent oxidation of glucose-6-phospate, which leads to the conversion of a nearly colorless probe to an intensely colored product with an absorbance at 450 nm. All assays were performed at 37 °C. NADP^+^/NADPH ratios in cell lines treated with PD were measured according to the protocol of NADP^+^/NADPH Quantification Kit (MAK038, Sigma). According to the NADPH standards, the concentration of NADPtotal or NADPH can be expressed in pmole per 10^6^ cells. The ratio of NADP^+^/NADPH was calculated by ((NADPtotal)–(NADPH)/(NADPH)).

### Cell viability assay

Cell viability was measured by the colorimetric 3-(4,5-dimethyl-2-thiazolyl)-2,5-diphenyltetrazolium bromide (MTT) assay. Cells were seeded in 96-well plates at a density of 10^4^ cells per well, then they were treated with 100 μL of 1 mg/mL MTT (Sigma) in DMEM medium containing 10% FBS for 4 h at 37 °C. The medium was then replaced with 200 μL of DMSO and shaken for 15 min, then absorbance at 540 nm was measured using a microplate ELISA reader with DMSO used as the blank. To quantify the synergistic or antagonist effect of the drugs combinations, CompuSyn software was used^43^.

### IF staining

After 24 h treatment with PD at various concentrations, cells were washed in PBS and fixed with 4% paraformaldehyde solution and permeabilized with 0.1% Triton X/PBS solution, then was performed a blocking in 1% BSA for 1 h at RT. Cells were incubated with anti-pIRE (Abcam, Cambridge, UK) in PBS for 30 min. Secondary antibodies were added after a PBS wash in the same conditions. Cells were incubated in a 1:500 solution of 10 mg/mL Hoechst (Invitrogen) in PBS for 10 min in the dark. To stain ER cells were incubated with 200 nM ER-Tracker Blue-White DPX in PBS solution for 20 min at 37°C. For positive control cells were exposed for 16 h to 5 μg/mL tunicamycin. Images were collected under a fluorescence microscope (EVOS FL Cell Imaging System, Thermo Scientific, Rockford, USA).

### CellROX assay

Cells were plated on glass bottom 35-mm MatTek dishes and treated with PD for 24 h or 100 μM menadione for 1 h at 37 °C. A quantity of 50 μM *N*-acetylcysteine was added menadione-treated wells. The cells were then stained with 5 μM CellROX green reagent by adding the probe to the complete media and incubating at 37 °C for 30 min. The cells were then washed with PBS and then imaged on a fluorescence microscope EVOS FL Cell Imaging System (Thermo Scientific, Rockford, USA). *N*-acetylcysteine treatment inhibited the fluorescent signal induced by menadione, confirming that the signal was specifically produced by ROS increase^44, 45^.

### FACS analysis

Apoptosis (Annexin V apoptosis detection kit, BD biosciences), CellROX assay (Thermo Fisher Scientific, USA), were performed according to the manufacturer’s instructions. Cells were analyzed with a FACSAria III (BD Biosciences, San Jose, CA) or a BD Accuri Cytometer (BD Biosciences, San Jose, CA). Data were analyzed by FlowJo V10 software (FlowJo LLC, USA). For cell cycle analysis, cells were detached from the plates by trypsinization and then fixed with ice-cold 80% ethanol. The cells were centrifuged and then stained with a solution of 50 µg/mL propidium iodide and 80 µg/mL RNase A for 60 min at 4 °C in the dark. DNA content and cell cycle distribution were measured with a FACSARIA III or BD Accuri Cytometer, data were analyzed using Mod-Fit software (Verity Software House, USA).

### G6PD overexpression

p3-G6PD-t1 and negative control pCMV3-untagged-NCV (control) hygroycin-resistant plasmids were purchased from Sino Biological Inc. (Sino Biological, Beijing, China). MCF7 cells were stably transfected with Lipofectamine 3000 (Thermo Fisher Scientific, Waltham, MA USA) according to the manufacturer’s instructions. Clones with upregulated expression of G6PD were selected with 100 µg/mL Hygromycin. Clones were screened by western blot.

### IRE1 and PERK downregulation

UMSCC103 cells were transfected with siRNAs targeting IRE1 (SASI_Hs01_00194923, Sigma), PERK (SASI_Hs0100096844, Sigma) or a control siRNA (SIC001, Sigma) using Lipofectamine 3000 (Thermo Fisher Scientific, Waltham, MA, USA) by following the manufacturer’s instructions.

### Protein extraction and western blotting

Cells were lysed in 1X RIPA buffer (150 mM NaCl, 1% NP-40, 0.5% sodium deoxycholate, 0.1% SDS, 50 mM Tris-HCl pH 7.5) plus 1% protease inhibitor cocktail, 1% PMSF (200 mM) and 1% sodium orthovanadate (Santa Cruz Biotechnology, USA). Lysates were clarified by centrifugation at 8000×*g* for 5 min at 4°C and equal amounts of protein were fractionated by SDS-PAGE and subsequently transferred onto nitrocellulose membrane, immunoblots were visualized using Supersignal® West Pico Chemiluminescent substrate (Thermo Scientific, Rockford, USA). Proteins were detected with anti-Glucose 6 Phosphate Dehydrogenase (Novus Biologicals, USA); anti-PERK (phospho T981) (#1055, Elabscience, Huston, USA); anti-PERK (#3667, Elabscience, Huston, USA); anti-IRE1 (phospho S724) (ab48187, Abcam, Cambridge, UK); anti-IRE1 (ab ab37073, Abcam, Cambridge, UK); Anti-eIF2α (phosphor S51) (9721, Cell Signaling, USA); anti-α-Tubulin Antibody (#2144 Cell Signalling Technology, UK); Anti-Calreticulin (ab2907, Abcam, Cambridge, UK); Anti-Calnexin (ab22595, Abcam, Cambridge, UK); Anti-BiP (BD610978, BD Biosciences, San Jose, CA); Anti-ATF4 (sc-200, Santa Cruz, Dallas, USA); Anti-Chop (sc7351, Santa Cruz, Dallas, USA); Anti-XBP1 (ab198999, Abcam, Cambridge, UK) and Anti-GAPDH (ab9485, Abcam, Cambridge, UK) were used for assessing loading.

### Mass spectrometry

Samples (~50 µg protein) were reduced, alkylated, and prepared as previously described^46^. Samples (3 µL, ~ 5 µg protein digest) were directly injected by autosampler (Eksigent nanoLC 425 LC system) at 5 µL/min onto a YMC Triart-C_18_ column (15 cm, 3 µm, 300 µm i.d.) using gradient elution (2–40% mobile phase B, followed by wash at 80% B and re-equilibration) over either 73 (120 min run time) min (for spectral library construction using data/information dependent acquisition DDA/ IDA) or 43 min (60 min run time) for SWATH/DIA (data independent acquisition) analysis. Mobile phases consisted of A: water containing 0.1% (v/v) formic acid; B: acetonitrile containing 0.1% (v/v) formic acid. The LC system was hyphenated to a Sciex TripleTof 5600+ mass spectrometer fitted with a Duospray source and 50 µm electrode suitable for microflow proteomic analysis. The IDA method was run with parameters of: CUR 25; GS1 15; GS2 0; ISVF 5500; TEM 0. TOFMS mass range of 400–1250 m/z; accumulation time of 250 ms with product ion scans on the top 30 ions before switching (dynamic exclusion for 20 s) with rolling collision energy selected. Product ion accumulation time was set to 50 ms giving a cycle time of 1.8 s. The SWATH method was run with the same source parameters as the IDA with a 50 ms TOFMS scan followed by 100 variable SWATH windows (optimized on an IDA datafile of the same samples) of 25 ms between 100 and 1500 m/z giving a cycle time of 2.6 s.

A spectral library for SWATH data extraction was constructed using the output from ProteinPilot 5.0 (Sciex) searching against the Swissprot Human database (January 2015) with the addition of iRT peptides to the. fasta file, combining eight IDA runs (pooled samples) and filtered and aligned to spike in iRT peptides (Biognosys, Switzerland) using PeakView 2.1 (Sciex). SWATH data extraction, quantitation, and fold change analysis were carried out using Sciex’s OneOmics cloud processing software suite incorporating processing methodology from ref. ^47^. Proteins were considered as differentially expressed if they had quantitative data on more than a single peptide, with a OneOmics confidence threshold of 70%.

### In vivo experiments

Twenty Foxn1nu nude male mice (6–8 weeks old) were purchased from the Harlan, Italy (Sanpietro al Natisone). The mice were maintained under pathogen-free conditions with food and water ad libitum, on 12/12 h day/night cycle, a temperature of 20 ± 2 °C, ten mice per cage randomized according to weight and tumor burden and divided in two treatment groups respectively; control vehicle (ethanol 100%) and polydatin 100 mg/kg i.p. UMSCC103 cells were trypsinized, centrifuged, and then resuspended in serum-free medium. For implantation, tumor cells were orthotopically inoculated into the right lateral portion of the tongue of mice (1 × 10^6^ cells per mouse) using a syringe with a 30 gauge disposable needle (BD Biosciences). Animals were anesthetized with intraperitoneal injections of zolazepam, atropine and xilazine. From 10 days post injection, mice were treated three times a week with either control vehicle or polydatin 100 mg/kg via i.p. Tumor growth was assessed every 3 days, and cervical lymph node metastasis were evaluated with Ultrasound System Vevo 2100 (Visualsonics, Canada) at 10 and 20 days post cell injection. For each group the area of the cervical lymph node was assessed with the Vevo 2100 commercial software. At the end of the experiment (30 days of treatments), mice were killed by CO_2_ inhalation, dissected, and the tongue and cervical lymph nodes weighed and photographed. The experimental protocols were in compliance with the European Community Council directive (86/609/EEC).

## Electronic supplementary material

Supplementary Table 1

Supplementary Table 2

Supplementary Figure 1

Supplementary Figure 2

Supplementary Figure 3

Supplementary Figure 4

Supplementary Figure 5

supplementary figure legends
