# Supplementary material for: A new inhibitor of glucose-6-phosphate dehydrogenase blocks pentose phosphate pathway and suppresses malignant proliferation and metastasis in vivo
Source: Cell Death Dis. 2018 May 14;9(5):572. doi: 10.1038/s41419-018-0635-5 (PMC5951921; doi:10.1038/s41419-018-0635-5)
Supplement: Supplementary file 1 — Supplementary Table 1 [file 41419_2018_635_MOESM1_ESM.docx]

| **Protein** | | **Tunicamycin vs NT** | | **Polydatin vs NT** | |
| --- | --- | --- | --- | --- | --- |
|  |  | **Fold change** | **Confidence** | **Fold change** | **Confidence** |
| sp\|Q13751\|LAMB3_HUMAN | Laminin subunit beta-3 | 5,35 | 0,92 | - | - |
| sp\|Q9Y4L1\|HYOU1_HUMAN | Hypoxia up-regulated protein 1 | 1,62 | 0,88 | - | - |
| sp\|P41250\|SYG_HUMAN | Glycine--tRNA ligase | 1,57 | 0,87 | - | - |
| sp\|P07339\|CATD_HUMAN | Cathepsin D | -1,76 | 0,86 | - | - |
| sp\|P43490\|NAMPT_HUMAN | Nicotinamide phosphoribosyltransferase | 1,51 | 0,84 | - | - |
| sp\|P04818\|TYSY_HUMAN | Thymidylate synthase | -2,71 | 0,83 | - | - |
| sp\|Q16831\|UPP1_HUMAN | Uridine phosphorylase 1 | 1,82 | 0,82 | - | - |
| sp\|P49588\|SYAC_HUMAN | Alanine--tRNA ligase, cytoplasmic | 1,70 | 0,82 | - | - |
| sp\|P13667\|PDIA4_HUMAN | Protein disulfide-isomerase A4 | 1,46 | 0,81 | - | - |
| sp\|Q13753\|LAMC2_HUMAN | Laminin subunit gamma-2 | 1,60 | 0,81 | - | - |
| sp\|Q06210\|GFPT1_HUMAN | Glutamine--fructose-6-phosphate aminotransferase [isomerizing] 1 | 1,72 | 0,80 | - | - |
| sp\|P08238\|HS90B_HUMAN | Heat shock protein HSP 90-beta | -1,28 | 0,78 | - | - |
| sp\|P04183\|KITH_HUMAN | Thymidine kinase, cytosolic | -4,26 | 0,76 | - | - |
| sp\|P27797\|CALR_HUMAN | Calreticulin | 1,26 | 0,76 | - | - |
| sp\|P12004\|PCNA_HUMAN | Proliferating cell nuclear antigen | -1,31 | 0,75 | - | - |
| sp\|P55145\|MANF_HUMAN | Mesencephalic astrocyte-derived neurotrophic factor | 1,91 | 0,75 | - | - |
| sp\|P14625\|ENPL_HUMAN | Endoplasmin | 1,49 | 0,93 | - | - |
| sp\|P08195\|4F2_HUMAN | 4F2 cell-surface antigen heavy chain | 1,64 | 0,72 | 1,74 | 0,88 |
| sp\|P11021\|GRP78_HUMAN | 78 kDa glucose-regulated protein | 5,04 | 0,99 | 1,34 | 0,80 |
| sp\|P08243\|ASNS_HUMAN | Asparagine synthetase [glutamine-hydrolyzing] | 2,45 | 0,95 | 2,06 | 0,92 |
| sp\|Q9Y617\|SERC_HUMAN | Phosphoserine aminotransferase | 1,58 | 0,73 | 2,01 | 0,87 |
| sp\|P19012\|K1C15_HUMAN | Keratin, type I cytoskeletal 15 | -1,93 | 0,91 | -1,85 | 0,86 |
| sp\|P23381\|SYWC_HUMAN | Tryptophan--tRNA ligase, cytoplasmic | 1,64 | 0,89 | 1,58 | 0,84 |
| sp\|P05120\|PAI2_HUMAN | Plasminogen activator inhibitor 2 | 1,28 | 0,79 | -1,93 | 0,94 |
| sp\|P52292\|IMA1_HUMAN | Importin subunit alpha-1 | -2,02 | 0,79 | -1,47 | 0,69 |
| sp\|P07602\|SAP_HUMAN | Prosaposin | -1,32 | 0,76 | 1,47 | 0,82 |
| sp\|O75874\|IDHC_HUMAN | Isocitrate dehydrogenase [NADP] cytoplasmic | - | - | 1,77 | 0,88 |
| sp\|P11413\|G6PD_HUMAN | Glucose-6-phosphate 1-dehydrogenase | - | - | 1,94 | 0,89 |
| sp\|P52209\|6PGD_HUMAN | 6-phosphogluconate dehydrogenase | - | - | 1,47 | 0,85 |
| sp\|P47895\|AL1A3_HUMAN | Aldehyde dehydrogenase family 1 member A3 | - | - | -1,52 | 0,85 |
| sp\|O60701\|UGDH_HUMAN | UDP-glucose 6-dehydrogenase | - | - | 1,34 | 0,76 |
| sp\|Q16881\|TRXR1_HUMAN | Thioredoxin reductase 1, cytoplasmic | - | - | 1,88 | 0,75 |
| sp\|Q8TC12\|RDH11_HUMAN | Retinol dehydrogenase 11 | - | - | 2,09 | 0,81 |
| sp\|P30043\|BLVRB_HUMAN | Flavin reductase (NADPH) | - | - | 1,70 | 0,70 |
| sp\|P09758\|TACD2_HUMAN | Tumor-associated calcium signal transducer 2 | - | - | 2,89 | 0,96 |
| sp\|Q9NPH2\|INO1_HUMAN | Inositol-3-phosphate synthase 1 | - | - | 2,04 | 0,93 |
| sp\|P04792\|HSPB1_HUMAN | Heat shock protein beta-1 | - | - | 1,52 | 0,90 |
| sp\|P17844\|DDX5_HUMAN | Probable ATP-dependent RNA helicase DDX5 | - | - | -1,36 | 0,87 |
| sp\|Q9NZN4\|EHD2_HUMAN | EH domain-containing protein 2 | - | - | 1,77 | 0,86 |
| sp\|Q16850\|CP51A_HUMAN | Lanosterol 14-alpha demethylase | - | - | 2,32 | 0,83 |
| sp\|Q13501\|SQSTM_HUMAN | Sequestosome-1 | - | - | 2,75 | 0,82 |
| sp\|P02538\|K2C6A_HUMAN | Keratin, type II cytoskeletal 6A | - | - | -2,27 | 0,82 |
| sp\|Q03169\|TNAP2_HUMAN | Tumor necrosis factor alpha-induced protein 2 | - | - | 1,49 | 0,80 |
| sp\|Q9NR30\|DDX21_HUMAN | Nucleolar RNA helicase 2 | - | - | -1,57 | 0,80 |
| sp\|P00966\|ASSY_HUMAN | Argininosuccinate synthase | - | - | 1,66 | 0,79 |
| sp\|Q13641\|TPBG_HUMAN | Trophoblast glycoprotein | - | - | 2,13 | 0,78 |
| sp\|P63261\|ACTG_HUMAN | Actin, cytoplasmic 2 | - | - | 1,22 | 0,78 |
| sp\|P04843\|RPN1_HUMAN | Dolichyl-diphosphooligosaccharide--protein glycosyltransferase subunit 1 | - | - | 1,54 | 0,73 |
| sp\|P62851\|RS25_HUMAN | 40S ribosomal protein S25 | - | - | -1,29 | 0,73 |
| sp\|Q9P1F3\|ABRAL_HUMAN | Costars family protein ABRACL | - | - | 1,38 | 0,73 |
| sp\|P26373\|RL13_HUMAN | 60S ribosomal protein L13 | - | - | -1,28 | 0,72 |
| sp\|P62424\|RL7A_HUMAN | 60S ribosomal protein L7a | - | - | -1,24 | 0,71 |
| sp\|Q96HR9\|REEP6_HUMAN | Receptor expression-enhancing protein 6 | - | - | 1,99 | 0,70 |
| sp\|P08107\|HSP71_HUMAN | hsp71 | - | - | 1,27 | 0,70 |
| sp\|P48507\|GSH0_HUMAN | Glutamate--cysteine ligase regulatory subunit | - | - | 1,65 | 0,69 |

**Supplementary Table 1. Protein fold changes as determined by SWATH-MS.**
