# Supplementary material for: A new inhibitor of glucose-6-phosphate dehydrogenase blocks pentose phosphate pathway and suppresses malignant proliferation and metastasis in vivo
Source: Cell Death Dis. 2018 May 14;9(5):572. doi: 10.1038/s41419-018-0635-5 (PMC5951921; doi:10.1038/s41419-018-0635-5)
Supplement: Supplementary file 2 — Supplementary Table 2 [file 41419_2018_635_MOESM2_ESM.docx]

**Supplementary Table 2**

**Primers sequences for RT-PCR**

| **Gene name** | **5’-sequence-3’** | |  |
| --- | --- | --- | --- |
| ***CHOP*** | ***Forward***  ***Reverse*** | **AGAACCAGGAAACGGAAACAGA**  **TCTCCTTCATGCGCTGCTTT** | |
| **XBP1** | ***Forward***  ***Reverse*** | **CAGCACTCAGACTACGTGCA**  **ATCCATGGGGAGATGTTCTGG** | |
| **sXBP1** | ***Forward***  ***Reverse*** | **CTGAGTCCGCAAGCAGGTGCAG**  **ATCCATGGGGAGATGTTCTGG** | |
| **Gapdh** | ***Forward***  ***Reverse*** | **GGAGTCAACGGATTTGGTCG**  **CTTCCCGTTCTCAGCCTTGA** | |
