# Supplementary figures and images for: A new inhibitor of glucose-6-phosphate dehydrogenase blocks pentose phosphate pathway and suppresses malignant proliferation and metastasis in vivo
Source: Cell Death Dis. 2018 May 14;9(5):572. doi: 10.1038/s41419-018-0635-5 (PMC5951921; doi:10.1038/s41419-018-0635-5)

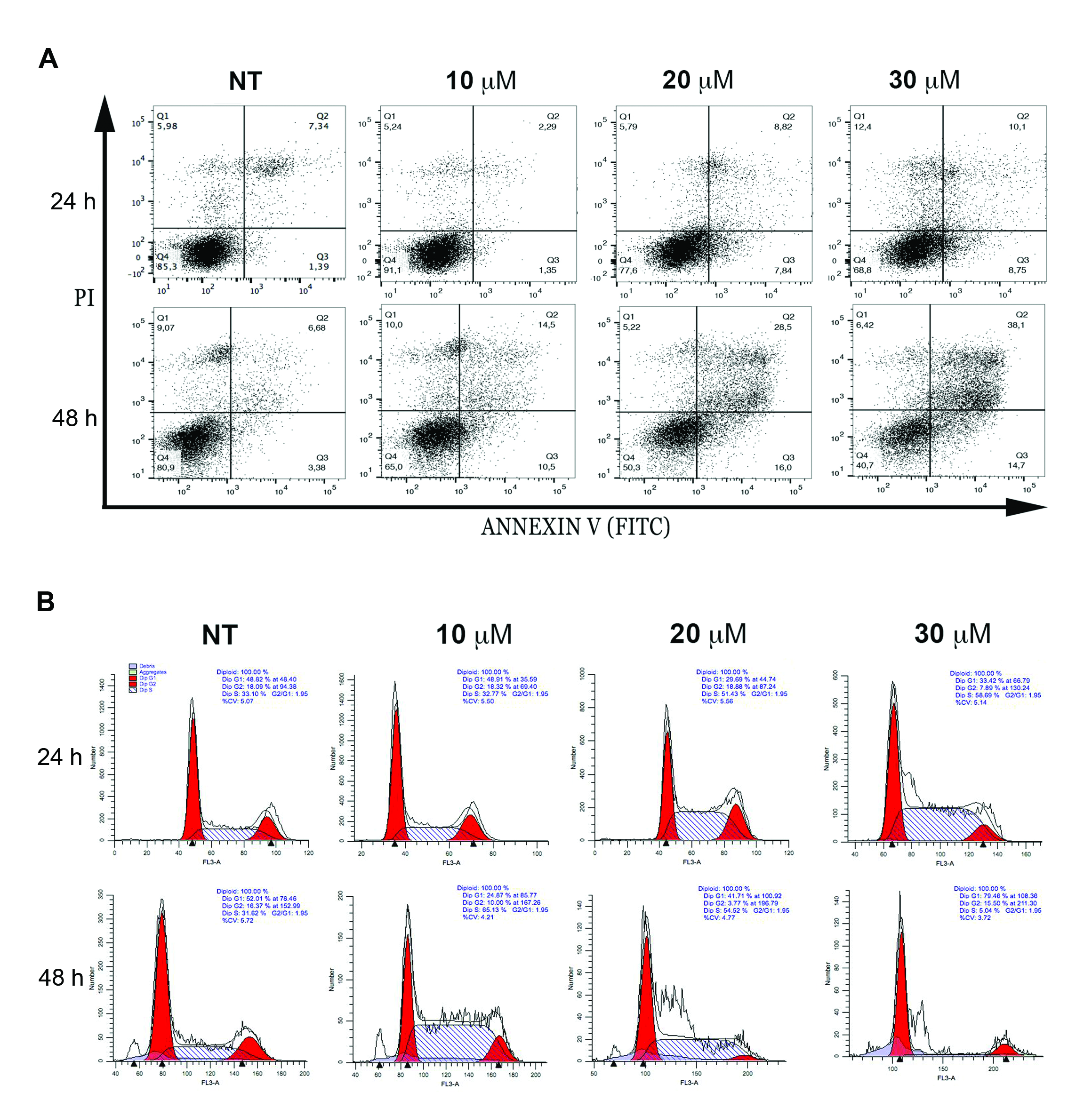

Supplement: Supplementary file 3 — Supplementary Figure 1 [file 41419_2018_635_MOESM3_ESM.tif]

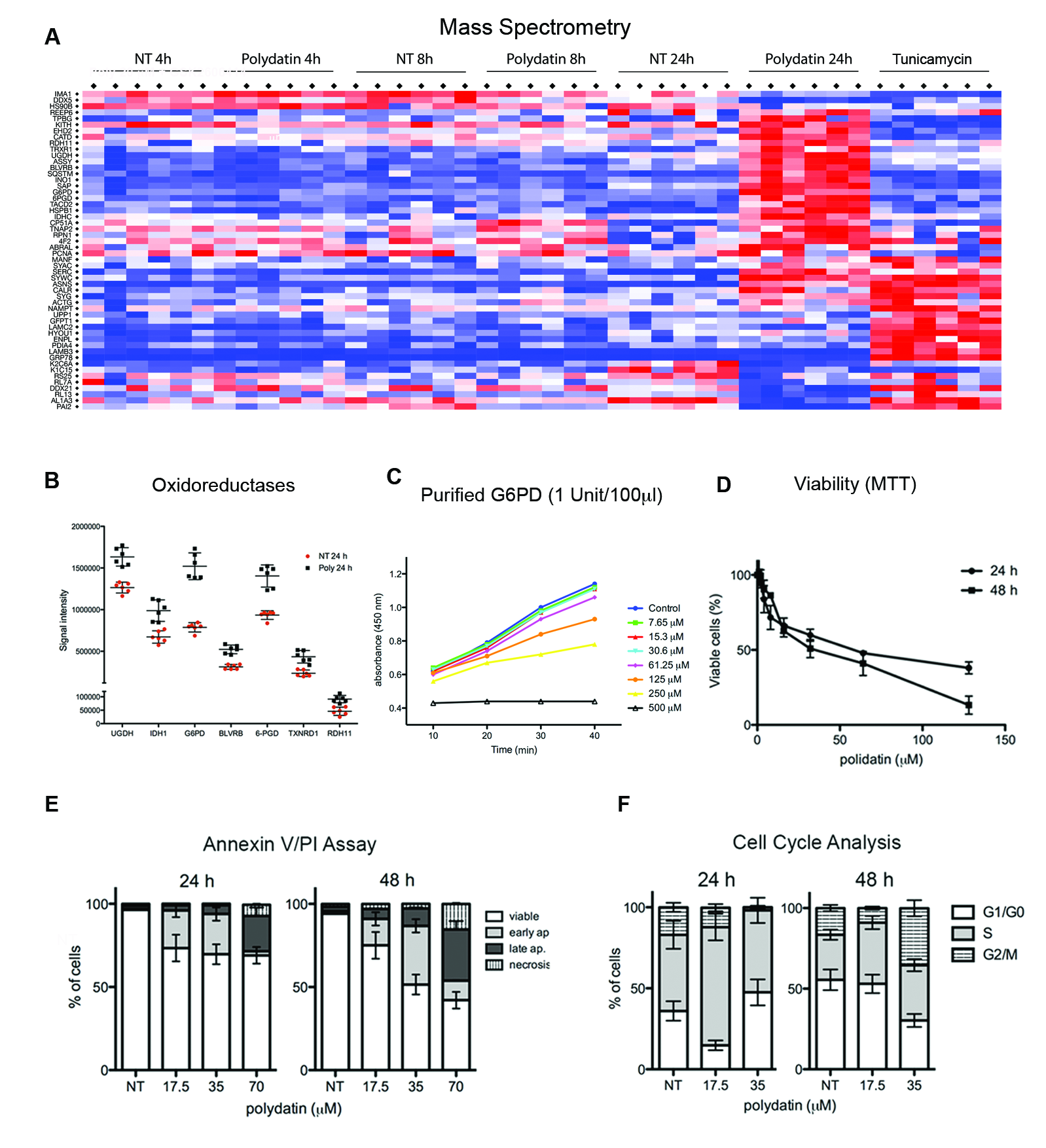

Supplement: Supplementary file 4 — Supplementary Figure 2 [file 41419_2018_635_MOESM4_ESM.tif]

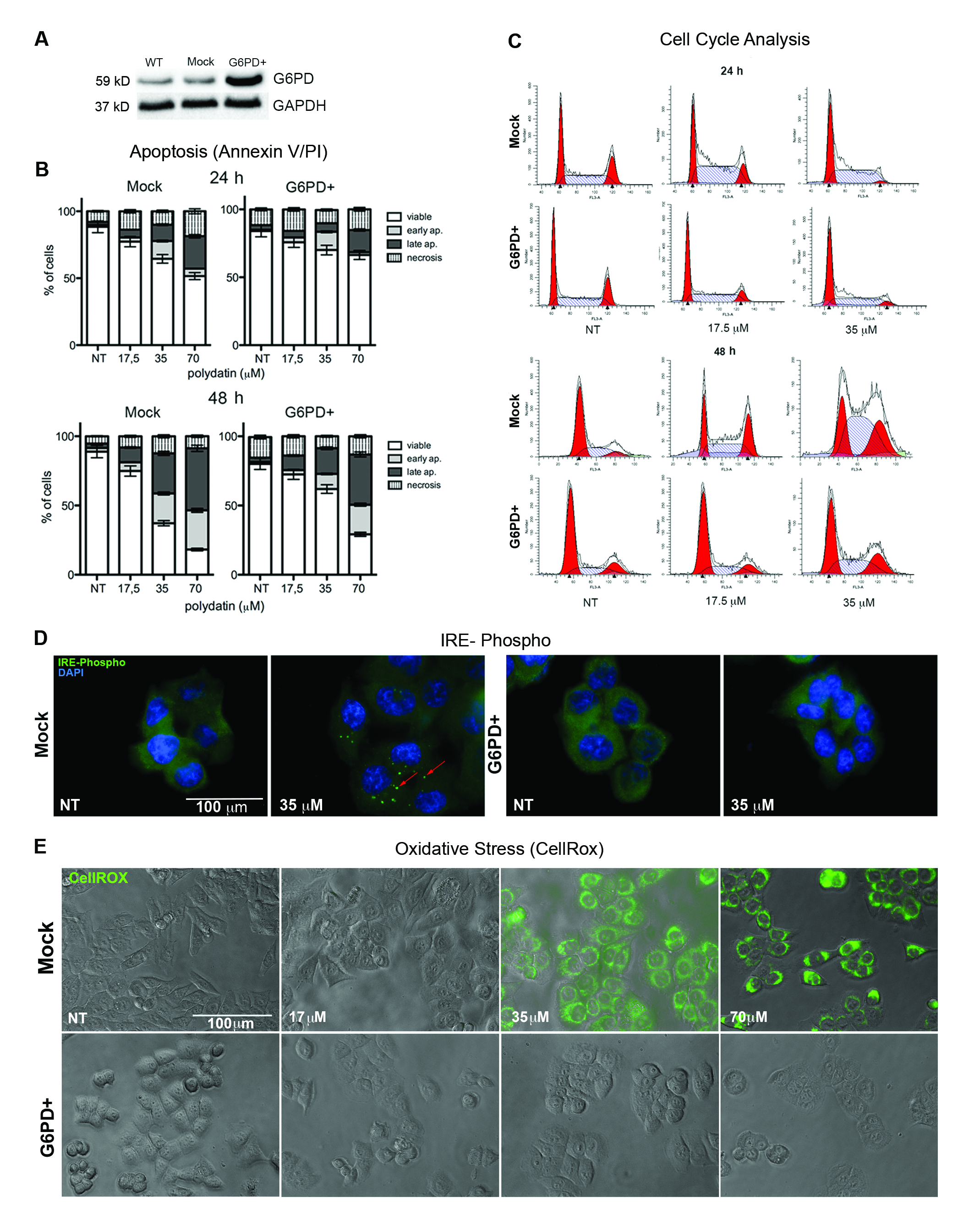

Supplement: Supplementary file 5 — Supplementary Figure 3 [file 41419_2018_635_MOESM5_ESM.tif]

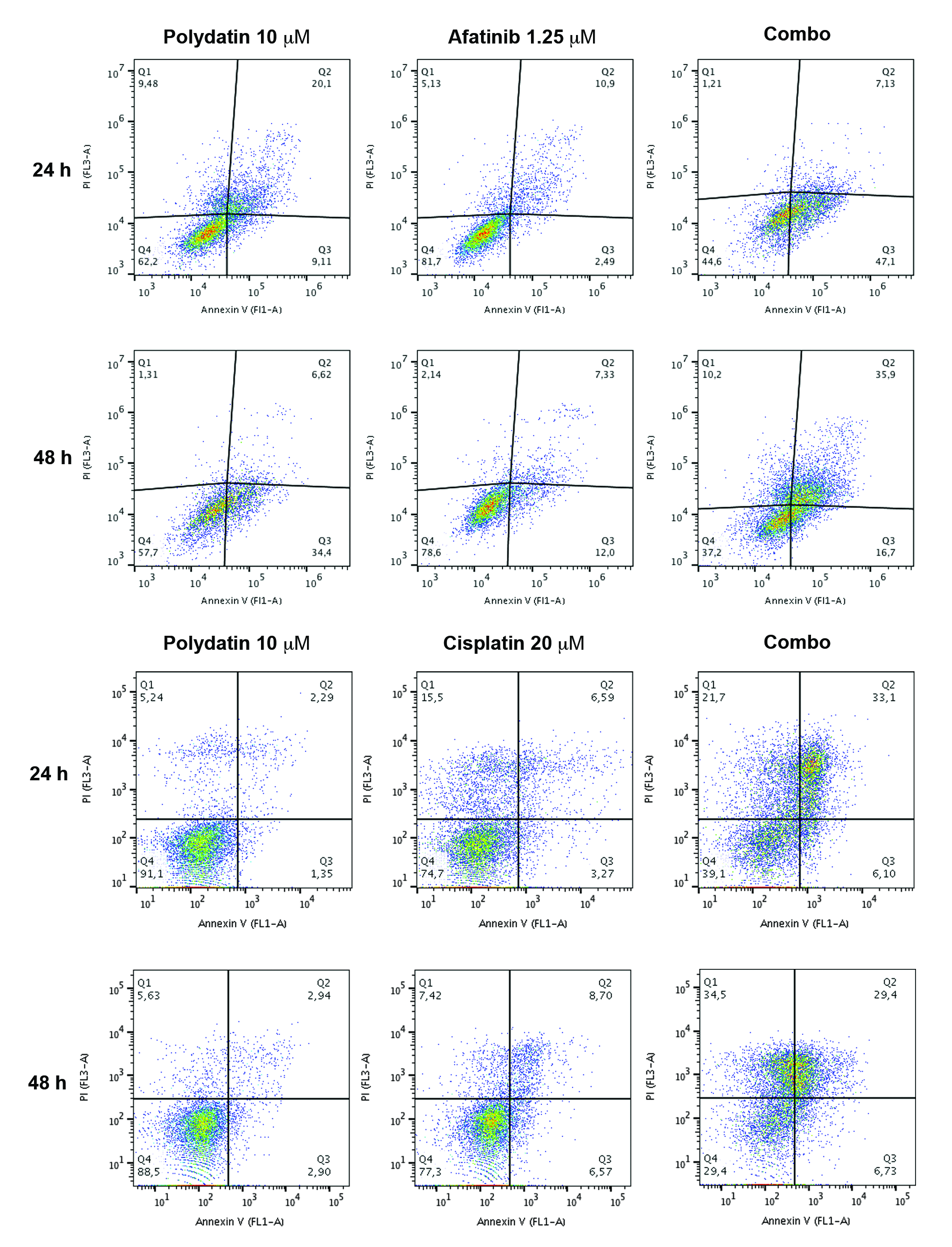

Supplement: Supplementary file 6 — Supplementary Figure 4 [file 41419_2018_635_MOESM6_ESM.tif]

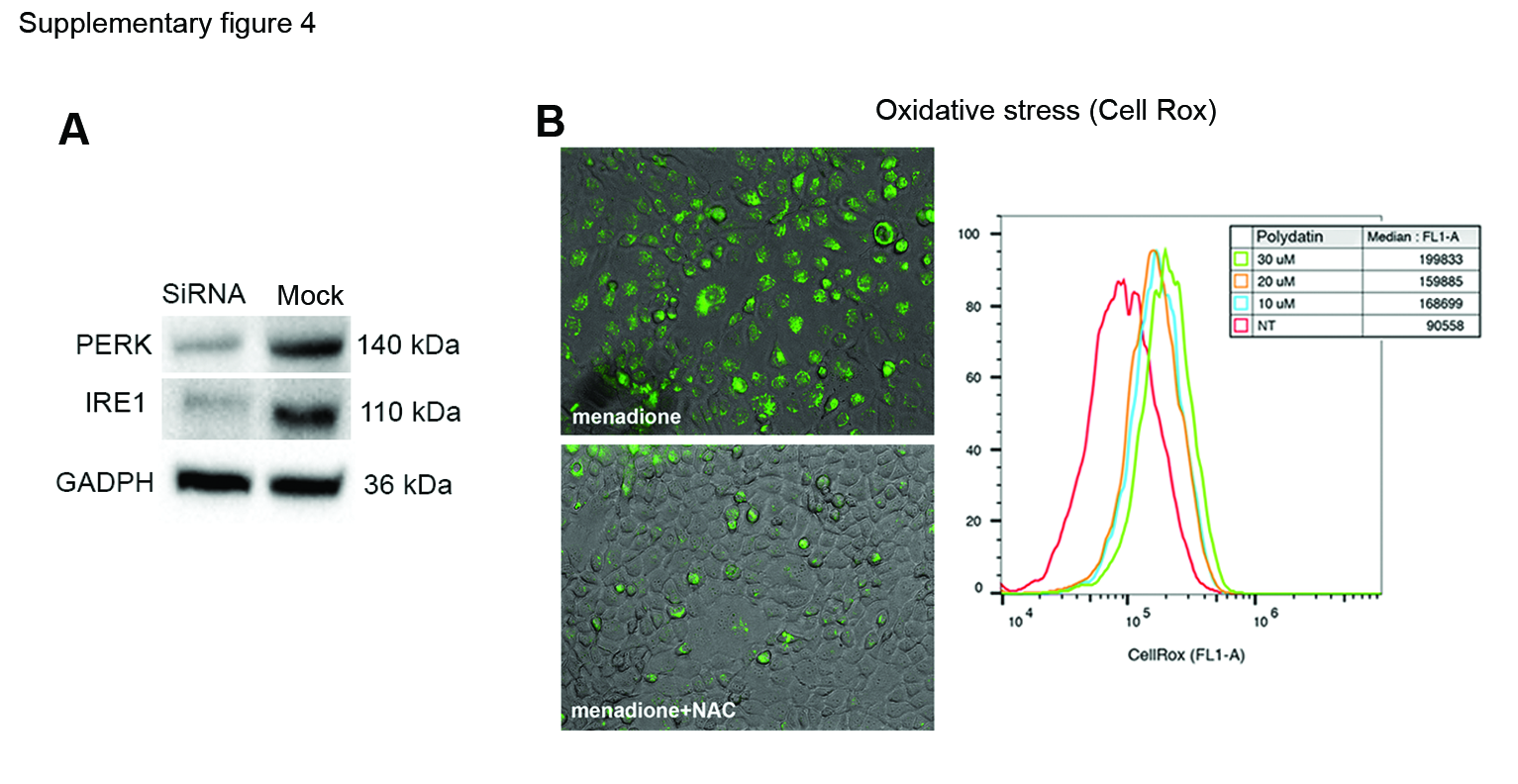

Supplement: Supplementary file 7 — Supplementary Figure 5 [file 41419_2018_635_MOESM7_ESM.tif]
