# Supplementary material for: A new inhibitor of glucose-6-phosphate dehydrogenase blocks pentose phosphate pathway and suppresses malignant proliferation and metastasis in vivo
Source: Cell Death Dis. 2018 May 14;9(5):572. doi: 10.1038/s41419-018-0635-5 (PMC5951921; doi:10.1038/s41419-018-0635-5)
Supplement: Supplementary file 8 — supplementary figure legends [file 41419_2018_635_MOESM8_ESM.docx]

**Supplementary Fig 1.**

**A)** Annexin V/PI flow cytometry dot plots of UMSCC103 treated with polydatin (same data as summarized in Fig. 1B). **B**) Flow cytometry histograms of cell cycle analysis with ModFit software (same data as summarized in Fig. 1C).

**Supplementary fig. 2. Mass Spectrometry, G6PD enzymatic assay and effect of polydatin on MCF7 cells.**

**A)** Heatmap showing relative protein abundance from SWATH-mass spectrometry performed on UMSCC103 treated with polydatin (20 µM); tunicamycin is used as positive control from ER stress related protein. **B)** Differential expression of oxidoreductases (mass spectrometry) between not treated (NT) and polydatin-treated cells (20 µM, 24 h after treatment). **C)** G6PD enzymatic assay performed with purified enzyme and polydatin. **D)** Viability assay (MTT) in MCF7 cells following polydatin treatment. **E)** Analysis of apoptosis on MCF7 cell line by Annexin V/PI assay at 24 and 48 h after treatment. **F)** Cell cycle analysis on MCF7 at 24 h and 48 h after treatment.

**Supplementary fig 3. Differences between G6PD+ and mock transfection following polydatin treatment.**

**A)** Viability assay (MTT) in G6PD+ and mock-transfected cells following polydatin treatment. **B)** Analysis of apoptosis by Annexin V/PI on G6PD+ and mock-transfected cells following polydatin treatment. **C)** Cell cycle analysis on G6PD+ and mock-transfected cells following polydatin treatment. **D**) IF for phospho-IRE in G6PD+ and mock-transfected cells following polydatin treatment (35 µM). **E)** IF of CellRox (oxidative stress determination) on G6PD+ and mock transfected cells following polydatin treatment. * p<0.05, N=3, error bar = 95% confidence

**Supplementary fig. 4. Polydatin increase the effect of cisplatin and afatininb.**

Annexin V/PI on UMSCC103 treated with polydatin and combination of polydatin and cisplatin or afatinib.

**Supplementary fig. 5. Additional data**

A) Immunoblot for IRE1 and PERK on cells transfected with siRNAs of mock vector. **B)** control images of CellRox IF shown in fig. 2; Flow cytometry histogram of CellRox after polydatin treatment. **C)** * p<0.05, N=3, error bar = 95% confidence
